# The Association of Uremic Toxins and Inflammation in Hemodialysis Patients

**DOI:** 10.1371/journal.pone.0102691

**Published:** 2014-07-22

**Authors:** Heng-Jung Hsu, Chiung-Hui Yen, I-Wen Wu, Kuang-Hung Hsu, Chih-Ken Chen, Chiao-Yin Sun, Chia-Chi Chou, Chun-Yu Chen, Chi-Jen Tsai, Mai-Szu Wu, Chin-Chan Lee

**Affiliations:** 1 Division of Nephrology, Chang Gung Memorial Hospital, Keelung, Taiwan; 2 The Graduate Institute of Clinical Medical Sciences, Chang Gung University Medical College, Taoyuan School of Medicine, Taoyuan, Taiwan; 3 Department of Pediatrics, Taipei Medical University Hospital, Taipei, Taiwan; 4 Laboratory of Epidemiology, Department of Health Care Management, Chang Gung University, Taoyuan, Taiwan; 5 Division of Psychiatry, Chang Gung Memorial Hospital, Keelung, Taiwan; 6 Division of Nephrology, Taipei Medical University Hospital, Taipei, Taiwan; 7 Department of Internal Medicine, Taipei Medical University, Taipei, Taiwan; San Raffaele Hospital, Italy

## Abstract

**Background:**

Cardiovascular disease is the leading cause of mortality in hemodialysis patients and is associated with chronic inflammation. Elevation of uremic toxins, particular protein-bound uremic toxins, is a possible cause of hyper-inflammation in hemodialysis patients. But the association between uremic toxins and inflammatory markers in hemodialysis is still unclear.

**Methods:**

We conducted a cross-sectional study to evaluate the association of the serum uremic toxins and inflammatory markers in hemodialysis patients.

**Results:**

The uremic toxins were not associated with inflammatory markers- including high sensitivity C-reactive protein, IL(Interleukin) -1β, IL-6, tumor necrosis factor-α. In multiple linear regression, serum levels of total *p*-cresol sulfate (*P*CS) were independently significantly associated with serum total indoxyl sulfate (IS) (standardized coefficient: 0.274, *p*<0.001), and co-morbidity of diabetes mellitus (DM) (standardized coefficient: 0.342, *p*<0.001) and coronary artery disease (CAD) (standardized coefficient: 0.128, *p* = 0.043). The serum total *P*CS levels in hemodialysis with co-morbidity of DM and CAD were significantly higher than those without co-morbidity of DM and CAD (34.10±23.44 vs. 16.36±13.06 mg/L, *p*<0.001). Serum levels of total IS was independently significantly associated with serum creatinine (standardized coefficient: 0.285, *p*<0.001), total *P*CS (standardized coefficient: 0.239, *p* = 0.001), and synthetic membrane dialysis (standardized coefficient: 0.139, *p* = 0.046).

**Conclusion:**

The study showed that serum levels of total PCS and IS were not associated with pro-inflammatory markers in hemodialysis patients. Besides, serum levels of total *P*CS were independently positively significantly associated with co-morbidity of CAD and DM.

## Introduction

End stage renal disease (ESRD) patients had higher cardiovascular disease (CVD)-related and all-cause mortality. Hyper-inflammation in hemodialysis patients results in CVD [1], [2]. Pro-inflammatory cytokines are crucial to the inflammation associated with malnutrition and atherosclerosis in ESRD. Furthermore, it has been shown that high sensitivity C-reactive protein (hs-CRP) is elevated in patients with chronic kidney disease (CKD).

Uremic toxins can be classified into small water soluble uremic toxins, middle molecule uremic toxins, and protein bound uremic toxins. Due to substantial albumin binding, current dialysis modality could provide adequate removal of small water soluble uremic toxins and partial removal of middle molecule uremic toxins, but difficult in protein-bound uremic toxins [3]. *P*-cresol sulfate (*P*CS) and indoxyl sulfate (IS) are protein-bound uremic toxins and were associated with endothelial dysfunction also immune dysregulation in renal disease [4], [5]. The cytokines' clearance decreases as well as the chronic renal failure stage increases. Many inflammatory cytokines are classified between the uremic toxins in particular tumor necrosis factor (TNF)- α, Interleukin(IL)-6, IL-1β are considered middle molecules as well as β2 microglobulin [6]. However, the exact association between uremic toxins and inflammation in ESRD patients is still not fully understood. Hence, we conducted the cross-sectional study to evaluate the association between uremic toxins and inflammatory markers.

## Subjects and Methods

### Patients

**Figure 1:**
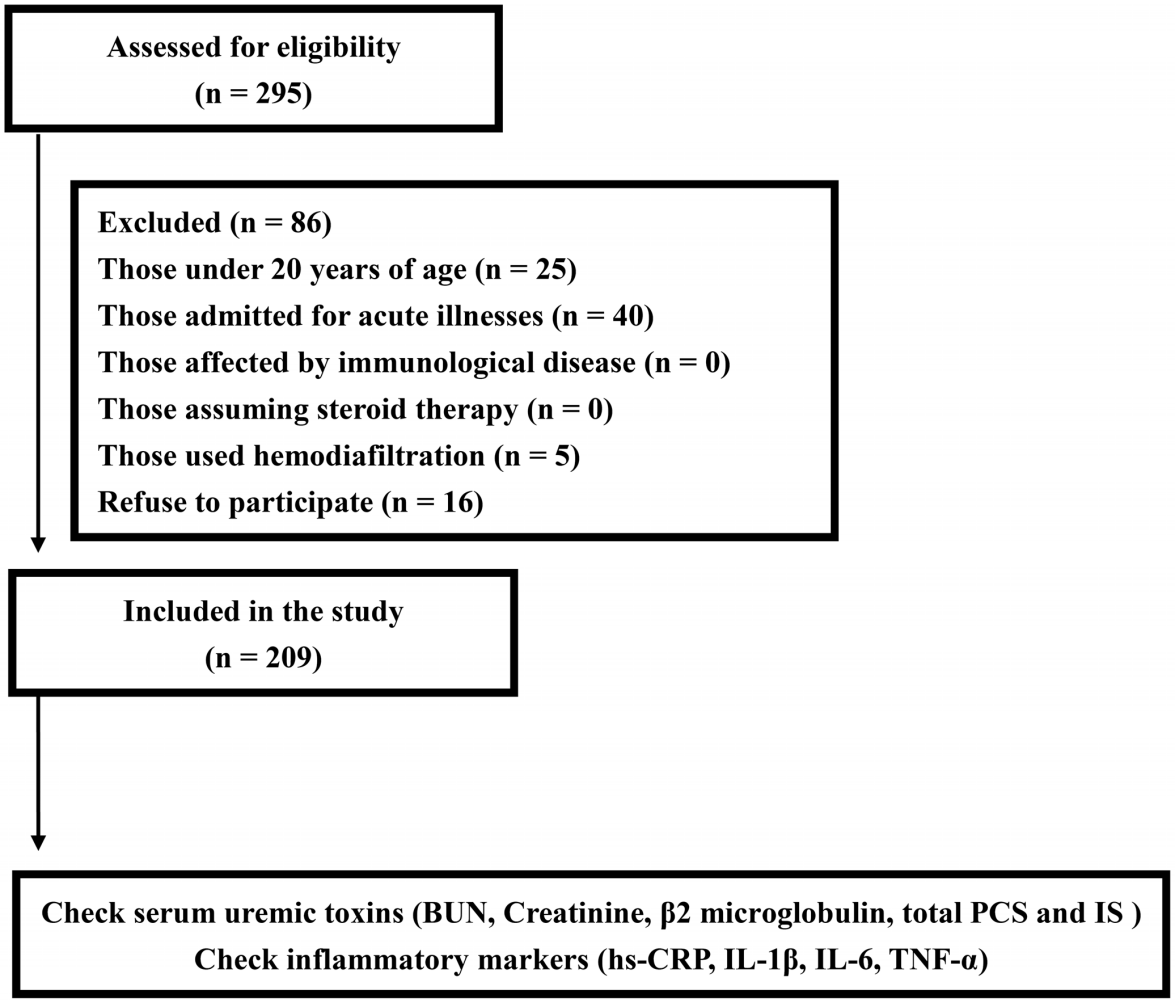
Flow chart indicates patient enrolment.

We conducted a cross-sectional study at the dialysis center of the Chang Gung Memorial Hospital, Keelung, Taiwan, enrolling 295 chronic hemodialysis patients who had been undergoing regular hemodialysis for more than 6 months. Excluded from the study were those patients who were under 20 years of age, admitted for acute illnesses, using hemodiafiltration, affected by immunologic disease, assuming steroid therapy, and had declined participation (Figure 1). All patients had been receiving a standardized hemodialysis regimen: dialysate flow, 500 mL/min; blood flow, 250–300 mL/min, duration of dialysis per session, 4 h; and, thrice-weekly hemodialysis. We screened the serum uremic toxins (blood urea nitrogen (BUN), creatinine, β2 microglobulin, total *P*CS and IS, and inflammatory markers (hs-CRP, IL-1β, IL- 6, TNF- α) in the study patients. The uremic toxins and cytokines were checked during the normal hemodialysis week. The information about dialysis membrane type, vascular access type, anticoagulation type, and angiotensin converting enzyme inhibitor (ACEI)/angiotensin II type 1 receptor blocker (ARB) assuming was recorded. All written informed consent for participation in the study was obtained from participants. This study complies with the Declaration of Helsinki and was approved by the Ethics Committee of the Institutional Review Board at Chang Gung Memorial Hospital.

### Measurement of the serum concentrations of total IS and PCS

To determine total IS and *P*CS serum levels, serum samples were deproteinized by adding 3 parts methanol to 1 part serum. All analyses were performed using a Waters Acquity Ultra Performance Liquid Chromatography (UPLC) system (Milford, MA, USA).

### Statistical Analysis

Continuous variables were expressed as means ± standard deviations. For normally distributed continuous variables, a two-tailed Student's unpaired *t*-test was employed to evaluate the differences between the means. The differences between groups of categorical variables were analyzed by the χ^2^ test or Fisher's exact test. To examine the relationship between IS/*P*CS and other variables, Pearson's correlation test was used. To identify independent associations, stepwise multiple regression analysis was used, and co-variants selection based on univariate analysis with variables with p<0.1 were selected. We considered *p*<0.05 statistically significant. All analyses were performed using SPSS version 17.0 for Windows.

## Results

### Study subjects

The mean age of the patients (99 men and 110 women) was 58.2±13.8 years old; while the mean duration of dialysis was 67.2±54.8 months (table 1). There were 75 (36% of the total study patients) diabetic patients in our study and all were type 2 diabetes mellitus (DM). All patients used anti-coagulation agents with 76.6% patients (n = 160) using heparin whereas 23.4% patients (n = 49) using low-molecular-weight heparin. There were 72.7% patients (n = 152) used arterial venous fistula as hemodialysis access. The prevalence of ACEI/ARB use in study patients was 34.4%. The biochemical and dialysis related parameters were shown in table 2. The serum levels of total IS and total *P*CS in the study patients were 36.70±16.74 and 21.25±17.81 mg/L, respectively.

**Table 1:** Baseline characteristics of study patients.

Characteristics	All patients
	N = 209
Age (years)	58.2±13.8
Male (%)	99 (47.4)
Smoking (%)	37 (17.7)
Diabetes (%)	75 (35.9)
CAD (%)	38 (18.2)
CHF (%)	43 (20.6)
PAOD (%)	26 (12.4)
COPD (%)	17 (8.1)
Stroke (%)	20 (9.6)
Cancer (%)	18 (8.6)
Dialysis vintage (months)	67.2±54.8
Body mass index (kg/m^2^)	23.3±3.9
nPCR (g/kg/day)	1.14±0.34
Cardiothoracic ratio (%)	0.50±0.07
Residual renal function (mL/min)	0.12±0.22
High flux dialysis (%)	137 (65.6)
Synthetic membrane (%)	160 (76.6%)
Vascular access	
AVF (%)	152 (72.7%)
AVG (%)	30 (14.4%)
Tunneled cuffed Catheter (%)	27 (12.9%)
ACEI/ARB use (%)	72 (34.4%)
Heparin use (%)	160 (76.6%)

Notes: Values are expressed as mean ± SD or total number (percent).

Abbreviations: CAD, coronary artery disease; CHF, congestive heart failure; PAOD, peripheral arterial occlusive disease; COPD, chronic obstructive pulmonary disease; nPCR, normalized protein catabolic rate; AVF, Arterial venous fistula; AVG, Arterial venous graft; ACEI, angiotensin converting enzyme inhibitor; ARB, angiotensin II type 1 receptor blocker.

**Table 2:** Biochemical and dialysis-related parameters of study patients.

Characteristics	All patients
	N = 209
Albumin (g/dL)	3.8±0.39
Hemoglobin (g/dL)	10.4±1.5
Calcium (mg/dL)	9.4±0.9
Phosphate (mg/dL)	5.2±1.8
Cholesterol (mg/dL)	177.1±47.7
Kt/V	1.67±0.36
URR (%)	0.74±0.07
BUN (mg/dL)	69.8±22.2
Creatinine (mg/dL)	10.8±2.7
β2 microglobulin (ng/mL)	28.3±11.5
i-PTH (pg/mL)	380.7±558.2
Total IS (mg/L)	36.70±16.74
Total *P*CS (mg/L)	21.25±17.81
hs-CRP (mg/L)	10.8±25.9
IL-1β (ng/L)	1.37±0.83
IL-6 (ng/L)	3.68±3.89
TNF-α (ng/L)	16.67±66.28

Abbreviations: URR, urea reduction rate; BUN, blood urea nitrogen; i-PTH, intact parathyroid hormone; IS, indoxyl sulfate; *P*CS: *p*-cresol sulfate; hs-CRP, high sensitive C-reactive protein; IL-1β, interleukin- 1 beta; IL-6, interleukin 6; TNF-α, tumor necrosis factor- alpha.

### Relationship between hemodialysis type, uremic toxins, and inflammatory markers

The associations among hemodialysis type, uremic toxins, and inflammatory markers are shown in table 3. Vascular access type was not significantly associated with uremic toxins and inflammatory markers. Hemodialysis synthetic membrane use was significantly associated with serum total IS (correlation coefficient: 0.195, *p*<0.001). High flux dialysis was also significantly associated with serum total IS (correlation coefficient: 0.159, *p* = 0.024). However, hemodialysis synthetic membrane or high flux dialysis was not associated with inflammatory markers- hs-CRP, IL-1β, IL-6, and TNF-α. In protein-bound uremic toxins, serum IS was positively associated with serum total *P*CS (correlation coefficient: 0.292, *p*<0.001). However, these was no association between serum IS and inflammatory markers- hs-CRP, IL-1β, IL-6, and TNF-α. Serum *P*CS was positive associated with serum total IS and not associated with inflammatory markers- hs-CRP, IL-1β, IL-6, and TNF-α. In the inflammatory markers, serum hs-CRP was positively associated with serum IL-6 and TNF-α. Serum IL-6 was positively associated with serum hs-CRP and TNF-α. However, IL-1β was not associated with other inflammatory markers - hs-CRP, IL-6, and TNF-α.

**Table 3:** The correlations between hemodialysis type, uremic toxins, and inflammatory markers.

	Vascular access	Synthetic membrane use	High flux dialysis	Total IS	Total *P*CS	hs-CRP	IL-1β	IL-6	TNF-α
Vascular access	—	−0.125	−0.101	−0.122	−0.083	−0.093	−0.046	0.049	−0.043
Synthetic membrane use	−0.125	—	0.865***	0.195***	−0.029	−0.060	−0.016	−0.106	−0.082
High flux dialysis	−0.101	0.865***	—	0.159**	−0.052	−0.028	0.005	−0.065	0.089
Total IS (mg/L)	−0.122	0.195***	0.159**	—	0.292***	−0.011	0.013	−0.062	−0.062
Total *P*CS (mg/L)	−0.083	−0.029	−0.052	0.292***	—	0.039	−0.102	0.074	0.093
hs-CRP (mg/L)	−0.093	−0.060	−0.028	−0.011	0.039	—	−0.033	0.407***	0.264***
IL-1β (ng/L)	−0.046	−0.016	0.005	0.013	−0.102	−0.033	—	0.004	−0.022
IL-6 (ng/L)	0.049	−0.106	−0.065	−0.062	0.074	0.407***	0.004	—	0.478***
TNF-α (ng/L)	−0.043	−0.082	0.089	−0.062	0.093	0.264***	−0.022	0.478***	—

*p value<0.1.

** *p* value<0.05.

***p value<0.01.

Abbreviations: IS, indoxyl sulfate; *P*CS: *p*-cresol sulfate; hs-CRP, high sensitive C-reactive protein; IL-1β, interleukin −1 beta; IL-6, interleukin 6; TNF-α, tumor necrosis factor- alpha.

### Factors associated with serum levels of total PCS

In order to cognize the factor associated with serum levels of total *P*CS, univariate correlation analysis was performed first. The univariate analysis revealed that the total *P*CS levels were positively associated with the presence of DM (correlation coefficient: 0.322, *p*<0.001), coronary artery disease (CAD) (correlation coefficient: 0.213, *p* = 0.003), stroke (correlation coefficient: 0.172, *p* = 0.017), and serum levels of BUN (correlation coefficient: 0.173, *p* = 0.014) and hemoglobin (correlation coefficient: 0.169, *p* = 0.017) (table 4). Besides, there was a positive association between the serum levels of total *P*CS and IS (correlation coefficient: 0.292, *p*<0.001). There was also a trend of partial positive association between presence of peripheral arterial occlusive disease (PAOD) and serum total *P*CS. However, inflammatory cytokine levels were not significantly associated with the serum total *P*CS levels. Stepwise multiple regression analysis revealed that the presence of DM and CAD, and the serum levels of total IS were independent factors associated with the serum total *P*CS levels after adjustment of age, gender, and factors that significant in univariate analysis (comorbidity of PAOD and stroke, serum levels of BUN and hemoglobin). Besides, there was a trend of positive association between presence of stroke and serum total *P*CS after multiple regression analysis. Co-morbidity of PAOD lost the trend of association with the serum total *P*CS levels after multiple regression analysis.

**Table 4:** Factors associated with serum total *p*-cresol sulfate.

Variable	Units	Univariate Correlation (correlation coefficient)	*p*	Multiple regression analysis (standardized coefficients; Beta)	*p*
Age	1 year	0.120	0.090*	-	-
Male vs female	-	0.126	0.075*	-	-
Smoking (yes vs no)	-	0.094	0.195	-	-
diabetes (yes vs no)	-	0.322	<0.001***	0.342	<0.001***
CAD (yes vs no)	-	0.213	0.003**	0.128	0.043**
CHF (yes vs no)	-	0.048	0.508	-	-
PAOD (yes vs no)	-	0.137	0.058*	-	-
COPD (yes vs no)	-	0.112	0.121	-	-
Stroke (yes vs no)	-	0.172	0.017**	0.117	0.075*
Cancer (yes vs no)	-	−0.107	0.139	-	-
Dialysis vintage	1 month	−0.045	0.530	-	-
Body mass index	1 kg/m^2^	0.018	0.795	-	-
nPCR	1 g/kg/day	0.070	0.342	-	-
CT ratio	1%	0.067	0.379	-	-
BUN	1 mg/dL	0.173	0.014**	-	-
Creatinine	1 mg/dL	0.085	0.232	-	-
Hemoglobin	1 g/dL	0.169	0.017**	-	-
Albumin	1 g/dL	0.057	0.420	-	-
hs-CRP	1 mg/L	−0.035	0.631	-	-
Calcium	1 mg/dL	−0.079	0.263	-	-
Phosphate	1 mg/dL	0.015	0.838	-	-
i-PTH	1 pg/mL	−0.078	0.274	-	-
IL-1β	1 ng/L	−0.102	0.169	-	-
IL-6	1 ng/L	0.074	0.426	-	-
TNF-α	1 ng/L	0.093	0.197	-	-
Total IS	1 mg/L	0.292	<0.001***	0.274	<0.001***
Kt/V		−0.086	0.251	-	-
URR	1%	−0.077	0.297	-	-
High Flux dialysis (yes vs no)	-	−0.023	0.425	-	-

Abbreviations: CAD, coronary artery disease; CHF, congestive heart failure; PAOD, peripheral arterial occlusive disease; COPD, chronic obstructive pulmonary disease; nPCR, normalized protein catabolic rate; CT ratio, cardiothoracic ratio; BUN, blood urea nitrogen; hs-CRP, high sensitive C- reactive protein; i-PTH, intact parathyroid hormone; IL-1β, interleukin −1 beta; IL-6, interleukin 6; TNF-alpha, tumor necrosis factor-alpha; IS, indoxyl sulfate; URR, urea reduction rate.

*: *p* value<0.1.

**: *p* value<0.05.

***: *p* value<0.01.

### Factors associated with serum levels of total PCS according to age

To assess the association between age, uremic toxins and co-morbidity, the study patients were grouped into older patients (Age ≧59 years old) and younger patients (Age <59 years old) by median age. In older patient group, serum total *P*CS levels were positively associated with co-morbidity of DM, CAD, stroke and serum levels of total IS (table 5). However, presence of DM (standardized coefficient: 0.399, *p*<0.001), stroke (standardized coefficient: 0.190, *p* = 0.034) and serum total IS (standardized coefficient: 0.266, *p* = 0.003) were independent factors associated with serum total *P*CS after multiple regression analysis. In younger patient group, serum total *P*CS levels were positively associated with presence of PAOD, and serum BUN and total IS. After multiple regression analysis, co-morbidity of PAOD (standardized coefficient: 0.260, *p* = 0.008) and serum total IS levels (standardized coefficient: 0.315, *p* = 0.001) were independent factors associated with serum total *P*CS.

**Table 5:** Factors associated with serum total *p*-cresol sulfate according to age.

Variable	Units	Univariate Correlation (correlation coefficient)	*p*	Multiple regression analysis (standardized coefficients; Beta)	*p*
**All patients**					
Age	1 year	0.120	0.090*	-	-
Male vs female	-	0.126	0.075*	-	-
diabetes (yes vs no)	-	0.322	<0.001***	0.342	<0.001***
CAD (yes vs no)	-	0.213	0.003**	0.128	0.043**
PAOD (yes vs no)	-	0.137	0.058*	-	-
Stroke (yes vs no)	-	0.172	0.017**	0.117	0.075*
BUN	1 mg/dL	0.173	0.014**	-	-
Hemoglobin	1 g/dL	0.169	0.017**	-	-
Total IS	1 mg/L	0.292	<0.001***	0.274	<0.001***
**Older patients by Median (Age ≧59 years old) (N = 105)**					
Male vs female	-	0.141	0.157	-	-
diabetes (yes vs no)	-	0.405	<0.001***	0.399	<0.001***
CAD (yes vs no)	-	0.248	0.013**	-	-
PAOD (yes vs no)	-	0.060	0.553	-	-
Stroke (yes vs no)	-	0.269	0.007***	0.190	0.034*
BUN	1 mg/dL	0.180	0.070*	-	-
Hemoglobin	1 g/dL	0.171	0.086*	-	-
Total IS	1 mg/L	0.249	0.012**	0.266	0.003***
**Younger patients by Median (Age <59 years old) (N = 104)**					
Male vs female	-	0.116	0.253	-	-
diabetes (yes vs no)	-	0.154	0.140	-	-
CAD (yes vs no)	-	0.099	0.346	-	-
PAOD (yes vs no)	-	0.259	0.012**	0.260	0.008***
Stroke (yes vs no)	-	−0.010	0.923	-	-
BUN	1 mg/dL	0.203	0.044**	-	-
Hemoglobin	1 g/dL	0.147	0.147	-	-
Total IS	1 mg/L	0.355	<0.001***	0.315	0.001***

Abbreviations: CAD, coronary artery disease; PAOD, peripheral arterial occlusive disease; BUN, blood urea nitrogen; IS, indoxyl sulfate.

*: *p* value<0.1.

**: *p* value<0.05.

***: *p* value<0.01.

### Factors associated with serum levels of total IS

The analysis revealed that the total IS levels were positively associated with male gender (correlation coefficient: 0.153, *p* = 0.029), serum levels of BUN (correlation coefficient: 0.143, *p* = 0.041), creatinine (correlation coefficient: 0.311, p<0.001), and total *P*CS levels (correlation coefficient: 0.292, *p*<0.001) (table 6). Moreover, the serum total IS levels were also strongly associated with synthetic membrane use (correlation coefficient: 0.195, *p*<0.001) and high flux dialysis (correlation coefficient: 0.159, *p*<0.001). There was also a trend of partial positive association between presence of CAD and serum total IS. Then stepwise multiple regression analysis revealed that synthetic membrane use (standardized coefficient: 0.139, *p* = 0.046) and serum levels of creatinine (standardized coefficient: 0.285, *p*<0.001) and total *P*CS (standardized coefficient: 0.239, *p* = 0.001) were independent factors associated with the serum total IS levels. Inflammatory markers including hs-CRP and cytokine levels were not associated with the serum total IS levels. Co-morbidity of CAD, PAOD, and stroke was also not independently associated with the serum total IS levels.

**Table 6:** Factors associated with serum total indoxyl sulfate levels.

Variable	Units	Univariate Correlation (correlation coefficient)	*p*	Multiple regression analysis (standardized coefficients; Beta)	*p*
Age	1 year	0.001	0.988	-	-
Male vs female	-	0.153	0.029**	-	-
Smoking (yes vs no)	-	0.072	0.319	-	-
diabetes (yes vs no)	-	−0.096	0.182	-	-
CAD (yes vs no)	-	0.132	0.067*	-	-
CHF (yes vs no)	-	−0.048	0.507	-	-
PAOD (yes vs no)	-	−0.005	0.944	-	-
COPD (yes vs no)	-	−0.331	0.669	-	-
Stroke (yes vs no)	-	−0.080	0.264	-	-
Cancer (yes vs no)	-	−0.090	0.210	-	-
Dialysis vintage	1 month	−0.032	0.647	-	-
Body mass index	1 kg/m^2^	0.005	0.940	-	-
nPCR	1 g/kg/day	0.014	0.852	-	-
CT ratio	1%	−0.072	0.345	-	-
BUN	1 mg/dL	0.143	0.041**	-	-
Creatinine	1 mg/dL	0.311	<0.001***	0.285	<0.001***
Hemoglobin	1 g/dL	0.127	0.070*	-	-
Albumin	1 g/dL	0.119	0.091*	-	-
hs-CRP	1 mg/L	−0.139	0.050*	-	-
Calcium	1 mg/dL	0.027	0.701	-	-
Phosphate	1 mg/dL	0.026	0.713	-	-
i-PTH	1 pg/mL	0.083	0.239	-	-
IL-1β	1 ng/L	0.013	0.859	-	-
IL-6	1 ng/L	−0.062	0.498	-	-
TNF-α	1 ng/L	−0.062	0.392	-	-
Total *PCS*	1 mg/L	0.292	<0.001 ***	0.239	0.001***
Kt/V		−0.031	0.674	-	-
URR	1%	0.003	0.969	-	-
Synthetic membrane use (yes vs no)	-	0.195	<0.001***	0.139	0.046*
High Flux dialysis (yes vs no)	-	0.159	<0.001***	-	-

Abbreviations: CAD, coronary artery disease; CHF, congestive heart failure; PAOD, peripheral arterial occlusive disease; COPD, chronic obstructive pulmonary disease; nPCR, normalized protein catabolic rate; CT ratio, cardiothoracic ratio; BUN, blood urea nitrogen; hs-CRP, high sensitive C- reactive protein; i-PTH, intact parathyroid hormone; IL-1β, interleukin - 1 beta; IL-6, interleukin 6; TNF-alpha, tumor necrosis factor-alpha; *P*CS, *p*-cresol sulfate; URR, urea reduction rate.

*: *p* value<0.1.

**: *p* value<0.05.

***: *p* value<0.01.

### Factors associated with serum levels of total IS according to age

In older patient group (Age ≧59 years old), serum total IS levels were positively associated with serum creatinine and total *P*CS (table 7). Serum total *P*CS levels (standardized coefficient: 0.224, *p* = 0.021) were independent factors associated with serum total IS after multiple regression analysis. In younger patient group (Age <59 years old), serum total IS levels were positively associated with male gender, and serum levels of creatinine and total *P*CS, and synthetic membrane use. After multiple regression analysis, only serum total *P*CS (standardized coefficient: 0.274, *p* = 0.004) and synthetic membrane use (standardized coefficient: 0.241, *p* = 0.012) were independent factors associated with serum total IS.

**Table 7:** Factors associated with serum total indoxyl sulfate according to age.

Variable	Units	Univariate Correlation (correlation coefficient)	*p*	Multiple regression analysis (standardized coefficients; Beta)	*p*
**All patients**					
Age	1 year	0.001	0.988	-	-
Male vs female	-	0.153	0.029**	-	-
CAD (yes vs no)	-	0.132	0.067*	-	-
PAOD (yes vs no)	-	−0.005	0.944	-	-
Stroke (yes vs no)	-	−0.080	0.264	-	-
BUN	1 mg/dL	0.143	0.041**	-	-
Creatinine	1 mg/dL	0.311	<0.001***	0.285	<0.001***
Hemoglobin	1 g/dL	0.127	0.070*	-	-
Albumin	1 g/dL	0.119	0.091*	-	-
hs-CRP	1 mg/L	−0.139	0.050*	-	-
Total *PCS*	1 mg/L	0.292	<0.001 ***	0.239	0.001***
Synthetic membrane use (yes vs no)	-	0.195	<0.001***	0.139	0.046*
High Flux dialysis (yes vs no)	-	0.159	<0.001***	-	-
**Older patients by Median (Age ≧59 years old) (N = 105)**					
Male vs female	-	0.001	0.995	-	-
CAD (yes vs no)	-	0.093	0.356	-	-
PAOD (yes vs no)	-	−0.002	0.987	-	-
Stroke (yes vs no)	-	−0.058	0.563	-	-
BUN	1 mg/dL	0.096	0.336	-	-
Creatinine	1 mg/dL	0.258	0.009***	-	-
Hemoglobin	1 g/dL	0.064	0.520	-	-
Albumin	1 g/dL	0.126	0.204	-	-
hs-CRP	1 mg/L	0.010	0.920	-	-
Total *PCS*	1 mg/L	0.249	0.012**	0.224	0.021**
Synthetic membrane use (yes vs no)	-	0.106	0.286	-	-
**Younger patients by Median (Age <59 years old) (N = 104)**					
Male vs female	-	0.300	0.002***	-	-
CAD (yes vs no)	-	0.180	0.082*	-	-
PAOD (yes vs no)	-	−0.010	0.926	-	-
Stroke (yes vs no)	-	−0.110	0.290	-	-
BUN	1 mg/dL	0.186	0.063*	-	-
Creatinine	1 mg/dL	0.381	<0.001***	-	-
Hemoglobin	1 g/dL	0.172	0.088*	-	-
Albumin	1 g/dL	0.119	0.239	-	-
hs-CRP	1 mg/L	−0.072	0.486	-	-
Total *PCS*	1 mg/L	0.355	<0.001 ***	0.274	0.004***
Synthetic membrane use (yes vs no)	-	0.309	0.002 ***	0.241	0.012**

Abbreviations: CAD, coronary artery disease; PAOD, peripheral arterial occlusive disease; BUN, blood urea nitrogen; hs-CRP, high sensitive C- reactive protein; *P*CS, *p*-cresol sulfate.

*: p value<0.1.

**: p value<0.05.

***: p value<0.01.

### Comparison of serum levels of total PCS in hemodialysis patients with comorbidity of DM and CAD or not

**Figure 2:**
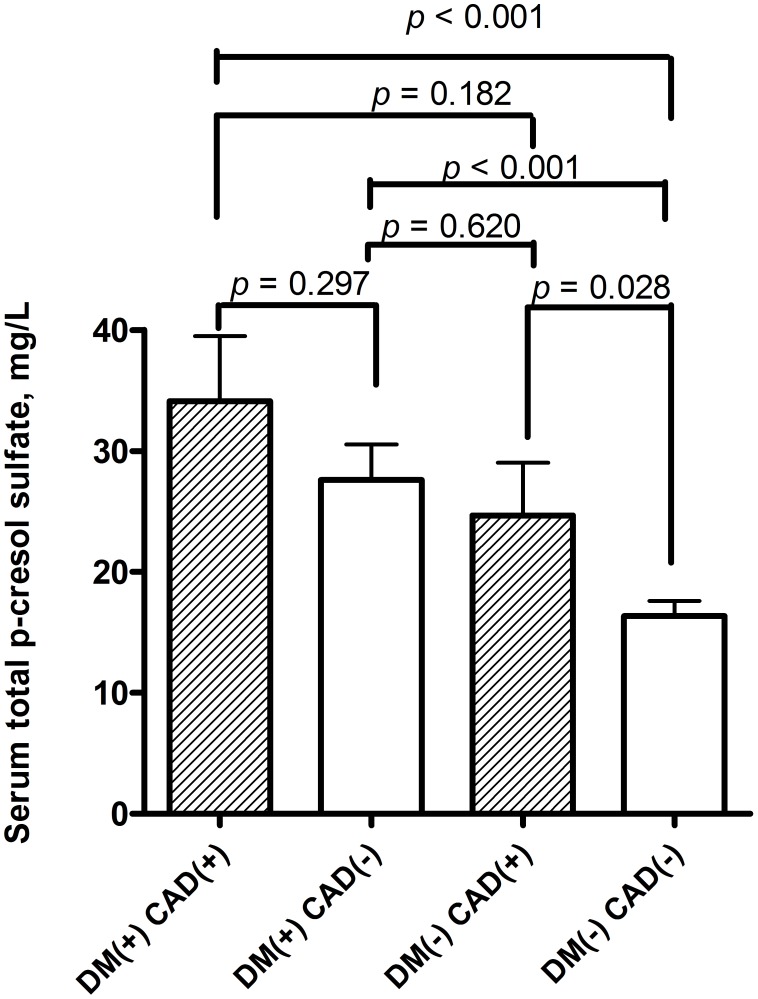
The serum levels of total *p*-cresol sulfate in hemodialysis patients with or without diabetes mellitus (DM) and coronary artery disease (CAD).

Due to co-morbidity of DM was independently associated with serum levels of total *P*CS. The baseline characteristics and also biochemical and dialysis-related parameters were also analysis according to the presence or absence of DM (table 8 and table 9). Those hemodialysis patients with DM were older, shorter dialysis vintage, with more cigarette smoking, higher prevalence of co-morbidity of CAD, PAOD, stroke, and lower serum level of creatinine, intact parathyroid hormone (i-PTH), Kt/V and urea reduction rate (URR). Besides, higher serum levels of total *P*CS were noted in those patients with diabetes than those without diabetes (DM patients vs. non DM patients: 29.36±21.59 vs. 17.38±13.77 mg/L, *p*<0.001). Furthermore, previous multiple linear regression analysis showed that comorbidity of CAD was also independently significantly associated with serum total *P*CS. The serum levels of total *P*CS were also compared between those patients with comorbidity of CAD or not. Those patients with comorbidity of CAD had higher serum levels of total *P*CS than those without CAD (CAD patients vs. non CAD patients: 29.94±21.02 vs. 19.97±16.75 mg/L, *p* = 0.003) (data also not shown in table). In order to configure out the association of serum total *P*CS and comorbidity of DM and CAD, patients were divided into four groups: those with comorbidity of DM and CAD; with comorbidity of DM but no CAD; with comorbidity of CAD but not DM; with comorbidity of neither DM nor CAD. We compared the serum total *P*CS levels between those four groups of patients. In hemodialysis patients with DM, the serum levels of *P*CS in those patients with CAD (n = 19) were higher than patients without CAD (n = 56) though without significance (34.10±23.44 vs. 27.60±20.83 mg/L, *p* = 0.297) (figure 2). In hemodialysis patients without DM, the serum levels of *P*CS in those patients with CAD (n = 19) were significantly higher than those patients without CAD (n = 115) (24.68±16.80 vs. 16.36±13.06 mg/L, *p* = 0.028). The serum levels of *P*CS in those patients with DM but not CAD (n = 56) were significantly higher than those neither DM nor CAD (n = 115) (27.60±20.83 vs. 16.36±13.06 mg/L, *p*<0.001). Besides, serum levels of total *P*CS in those patients with DM but not CAD (n = 56) were higher than those with CAD but not DM (n = 19): (27.60±20.83 vs. 24.68±16.80 mg/L, *p* = 0.620) though without significance. Moreover, serum levels of total *P*CS in those patients with DM and CAD (n = 19) were significantly higher than those with neither DM nor CAD (n = 115): (34.10±23.44 vs. 16.36±13.06 mg/L, *p*<0.001).

**Table 8:** Baseline characteristics according to the presence or absence of diabetes mellitus.

Characteristics	No diabetic	Diabetic	*p*
	N = 134	N = 75	
Age (years)	57.0±15.2	61.1±10.8	0.046*
Male (%)	60 (44)	39 (52)	0.299
Smoking (%)	18 (13)	19 (25)	0.013*
CAD (%)	17 (13)	21 (28)	0.011*
CHF (%)	23 (17)	20 (27)	0.16
PAOD (%)	11 (8)	15 (20)	0.021*
COPD (%)	8 (6)	9 (12)	0.164
Stroke (%)	7 (5)	13 (17)	0.007*
Cancer (%)	13 (10)	5 (7)	0.381
Dialysis vintage (months)	82.0±73.4	42.0±35.2	<0.001*
Body mass index (kg/m^2^)	23.2±3.6	23.6±4.1	0.683
nPCR (g/kg/day)	1.18±0.29	1.15±0.27	0.66
Cardiothoracic ratio (%)	0.49±0.07	0.51±0.06	0.058
Residual renal function (mL/min)	0.11±0.18	0.13±0.29	0.755
High flux dialysis (%)	93 (69)	44 (59)	0.107
Synthetic membrane (%)	93(74)	51(68)	0.377
Vascular access			0.152
AVF (%)	89 (71)	60 (80)	
AVG (%)	17 (14)	10 (13)	
Catheter (%)	20 (16)	5 (7)	
ACEI/ARB use (%)	42 (33)	29 (39)	0.444
Heparin use (%)	93 (74%)	61 (81)	0.223

Notes: Values are expressed as mean ± SD or total number (percent).

*: *p* value<0.05.

Statistical significance based on Chi-square test for categorical variables or *t*-test for continuous variables.

Abbreviations: CAD, coronary artery disease; CHF, congestive heart failure; PAOD, peripheral arterial occlusive disease; COPD, chronic obstructive pulmonary disease; nPCR, normalized protein catabolic rate; AVF, Arterial venous fistula; AVG, Arterial venous graft; ACEI, angiotensin converting enzyme inhibitor; ARB, angiotensin II type 1 receptor blocker.

**Table 9:** Biochemical and dialysis-related parameters according to the presence or absence of diabetes mellitus.

Characteristics	No diabetic	Diabetic	*p*
	N = 134	N = 75	
BUN (mg/dL)	70.8±24.1	68.8±18.7	0.55
Creatinine (mg/dL)	11.3±2.8	10.0±2.3	0.001*
Hemoglobin (g/dL)	10.5±1.6	10.3±1.3	0.271
Albumin (g/dL)	3.8±0.4	3.8±0.4	0.319
hs-CRP (mg/L)	9.3±19.7	9.8±16.3	0.432
Calcium (mg/dL)	9.5±1.0	9.4±0.9	0.316
Phosphate (mg/dL)	5.1±1.8	5.3±1.7	0.39
Cholesterol (mg/dL)	174.2±35.6	184.0±63.8	0.165
i-PTH (pg/mL)	447.6±667.6	260.6±307.3	0.025*
IL-1β (ng/L)	1.38±0.85	1.39±0.84	0.989
IL-6 (ng/L)	3.69±4.74	3.93±2.26	0.761
TNF-α (ng/L)	15.06±70.38	19.70±62.73	0.645
Total indoxyl sulfate (mg/L)	38.49±17.54	35.19±14.60	0.182
Total *p*-cresol sulfate (mg/L)	17.38±13.77	29.36±21.59	<0.001*
Kt/V	1.71±0.37	1.59±0.34	0.033*
URR (%)	0.75±0.074	0.72±0.07	0.006*

*: *p* value<0.05.

Statistical significance based on *t*-test for continuous variables.

Abbreviations: BUN, blood urea nitrogen; hs-CRP, high sensitive C-reactive protein; i-PTH, intact parathyroid hormone; IL-1β, interleukin −1 beta; IL-6, interleukin 6; TNF-alpha, tumor necrosis factor-alpha; URR, urea reduction rate.

### Comparison of serum levels of total IS in hemodialysis patients with comorbidity of DM and CAD or not

**Figure 3:**
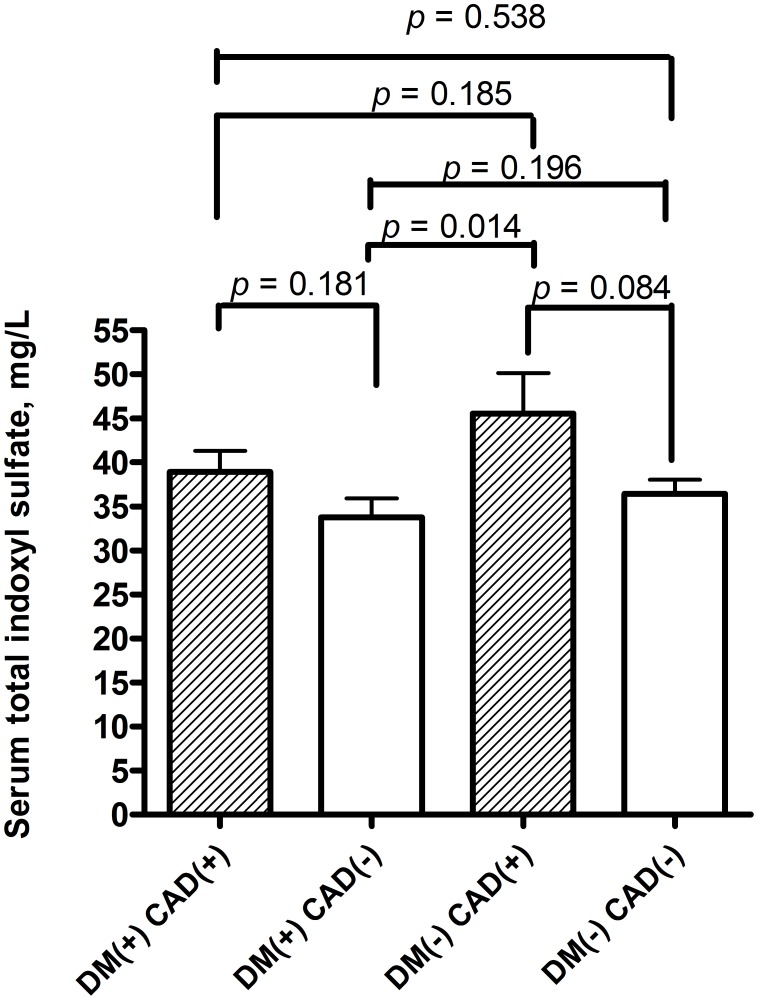
The serum levels of total indoxy sulfate in hemodialysis patients with or without diabetes mellitus (DM) and coronary artery disease (CAD).

The serum levels of total IS were compared between those patients with comorbidity of DM or not. Those hemodialysis patients with comorbidity of DM and those without diabetes had similar levels of total IS (DM patients vs. non DM patients: 35.19±14.60 vs. 38.49±17.54 mg/L, *p* = 0.182). Furthermore, the serum levels of total IS were also compared between those patients with comorbidity of CAD or not. Those patients with comorbidity of CAD had higher serum levels of total IS than those without CAD although without significance (CAD patients vs. non CAD patients: 41.86±14.82 vs. 36.25±16.81 mg/L, *p* = 0.067) (data also not shown in table). In order to configure out the association of serum total IS and comorbidity of DM and CAD, patients were also divided into four groups: those with comorbidity of DM and CAD; with comorbidity of DM but no CAD; with comorbidity of CAD but not DM; with comorbidity of neither DM nor CAD. In hemodialysis patients with DM, the serum levels of total IS in those patients with CAD (n = 19) were higher than patients without CAD (n = 56) (38.90±10.86 vs. 33.74±15.68 mg/L, *p* = 0.181) (figure 3). In hemodialysis patients without DM, the serum levels of total IS in those patients with CAD (n = 19) were higher than those patients without CAD (n = 115) (45.55±18.36 vs. 37.44±17.26 mg/L, *p* = 0.084). The serum levels of total IS in those patients with DM but not CAD (n = 56) were lower than those with neither DM nor CAD (n = 115) (33.74±15.68 vs. 37.44±17.26 mg/L, *p* = 0.196). Besides, serum levels of total IS in those patients with DM but not CAD (n = 56) were significantly lower than those with CAD but not DM (n = 19): (33.74±15.68 vs. 45.55±18.36 mg/L, *p* = 0.014). Moreover, serum levels of total IS in those patients with DM and CAD (n = 19) were lower than those CAD but not DM (n = 19): (38.90±10.86 vs. 45.55±18.36 mg/L, *p* = 0.185). Furthermore, previous univariate analysis found that serum levels of IS were associated with high flux dialysis. Biochemical and dialysis-related parameters were also compared between those patients with high flux and low flux dialysis. Those patients with high flux dialysis had higher serum levels of BUN, creatinine, hemoglobin, albumin, total IS, and URR (table 10).

**Table 10:** Biochemical and dialysis-related parameters according to high flux or low flux dialysis.

Characteristics	High flux	Low Flux	*p*
	N = 136	N = 70	
BUN (mg/dL)	72.7±21.7	64.3±22.3	0.009*
Creatinine (mg/dL)	11.5±2.6	9.6±2.4	<0.001*
Hemoglobin (g/dL)	10.7±1.6	10.0±1.2	0.003*
Albumin (g/dL)	3.9±0.4	3.8±0.4	0.008*
hs-CRP (mg/L)	8.9±19.1	10.0±16.1	0.695
Calcium (mg/dL)	9.5±0.9	9.2±0.9	0.018
Phosphate (mg/dL)	5.3±1.7	5.0±1.9	0.387
Cholesterol (mg/dL)	173.2±49.3	184.5±43.9	0.103
i-PTH (pg/mL)	422.5±646.8	299.5±311.6	0.134
IL-1β (ng/L)	1.38±0.86	1.37±0.78	0.943
IL-6 (ng/L)	3.50±3.64	4.03±4.34	0.478
TNF-α (ng/L)	21.03±80.74	8.75±21.67	0.211
Total indoxyl sulfate (mg/L)	38.60±15.94	33.00±17.75	0.024*
Total *p*-cresol sulfate (mg/L)	20.59±18.47	22.52±16.53	0.466
Kt/V	1.67±0.33	1.68±0.41	0.892
URR (%)	0.75±0.06	0.74±0.08	0.006*

*: *p* value<0.05.

Statistical significance based on *t*-test for continuous variables.

Abbreviations: BUN, blood urea nitrogen; hs-CRP, high sensitive C-reactive protein; i-PTH, intact parathyroid hormone; IL-1β, interleukin −1 beta; IL-6, interleukin 6; TNF-alpha, tumor necrosis factor-alpha; URR, urea reduction rate.

## Discussion

The mechanism of inflammation and higher CVD in ESRD patients under hemodialysis is multifactorial. Uremic toxins were considered one of possible cause to induce inflammation and CVD. In this study, the uremic toxins including small soluble, middle molecular, and protein-bound uremic toxins were all evaluated. The result showed that there was no significant association between above uremic toxins and inflammatory markers (hs-CRP, IL-1β, IL-6, and TNF-α). Furthermore, the serum levels of *P*CS were significantly higher in DM and CAD patients.

Malnutrition, inflammation, atherosclerosis syndrome is associated with an exceptionally high mortality rate in ESRD patients [2]. Elevations of several inflammatory markers including CRP, IL-6, and TNF-alpha were reported to be associated with mortality in ESRD patients undergoing hemodialysis or peritoneal dialysis [7], [8], [9], [10]. Although the causes of elevated serum levels of pro-inflammatory cytokines in ESRD patients are not well understood, reduced renal clearance of cytokines, accumulation of advanced glycation end-products (AGE), hyperhomocysteinemia [11], dialysis procedure- related inflammation [12], and unrecognized persistent infection were considered to induce the inflammation in ESRD patients. Uremic toxins, especially protein-bound uremic toxins, are also considered to cause inflammation [13], [14]. Protein-bound uremic toxins cannot be easily removed during dialysis procedure not matter HD or PD. In vitro studies had shown that *p*- cresol had toxic potential to endothelium. Clinical studies also showed significant association between serum *P*CS and CVD in CKD and ESRD patients [15], [16], [17], [18], [19]. Lee et al. found significant association between serum levels of free IS and IL-6 in peritoneal dialysis patients. But no significant association between serum levels of total IS and CRP, IL-6, and IL-10 was noted [20]. However, few studies exploded the association between uremic toxins and pro-inflammatory markers in hemodialysis patients. The study evaluated the uremic toxins including serum BUN, creatinine, β2 microglobulin, total *P*CS, and IS with association of pro-inflammatory cytokines in hemodialysis patients. The result showed lack association of uremic toxins and pro-inflammatory markers. Possible explanation is that inflammation induced by uremic toxins is transiently or focally, which cannot be observed by our study design. It is also possible that *P*CS directed induced atherosclerosis without via the cytokine and inflammation. The mechanism needs more studies to evaluate the close connection between total *P*CS and atherosclerosis in ESRD patients.

It is very interesting that serum total *P*CS was significantly associated with comorbidity of DM and also CAD. The exact connection between co-morbidity of DM, CAD, and serum total *P*CS was unclear. Because of the study design does not allow to establish any causal relationship between variable above that were examined. However, some working hypothesis can be considered. Possible hypotheses are that those patients with co-morbidity of CAD and DM had some particular factors that influence the levels of *P*CS production. But lots of evidences suggested that higher levels of *P*CS induced CAD by causing the deleterious effect on the endothelium. So the relationship between serum levels of total *P*CS and comorbidity of CAD could be understood and solved. But the association between serum total *P*CS and co-morbidity of DM is still unknown. Similarly, Meijers et al. also showed significant higher total and free *P*CS in hemodialysis patients with DM than non- DM [15]. Whether this is related to particular dietary habits is not clear. Indeed, protein intake is a limiting factor for the production by gut resident bacteria of *p* cresol. However, we can reasonable exclude this hypothesis because we did not observe any statistically significant difference among the study patients with DM or not in the serum albumin, BMI, and nPCR. Residual renal function and high flux hemodialysis were also important factors to determine protein-bound uremic toxins [21], [22]. However, the residual renal function and high flux dialysis use ratio did not significant between DM and non-DM patients (table 8). Another possible explanation is that patients with diabetes have a significant risk of developing severe constipation often due to dysfunction of the autonomic nervous system [23]. Some evidences suggests diabetic patients had altered colonic protein fermentation [24]. *P*-cresol was generated by the intestinal flora and then conjugated to *p*-cresol sulfate in the intestinal wall and to *p*-cresylglucuronide in the liver [3]. When bowel movement decreased due to autonomic dysfunction in DM patients, more gut formation and production of *P*CS may happen.

In hemodialysis patients with DM, although the difference was not prominent significance, there was a trend that the serum levels of total p-cresol sulfate were higher in those patients with co-morbidity of CAD than those without CAD. These result indicated that higher serum total PCS may be associated with CAD even in these high CVD risk patients (diabetic hemodialysis patients). The reason of no significance may be due to small patient number (patients with DM & CAD: n = 19 vs. DM without CAD: n = 56). In hemodialysis patients without DM, the serum levels of total *p*-cresol sulfate were significantly higher in those patients with co-morbidity of CAD than those without CAD. The results suggested that particular dietary habit or colonic protein fermentation in those HD patients may develop coronary artery disease no matter in DM or non-DM patients. In stratified analysis by age, serum levels of total *P*CS were independently significantly associated with comorbidity of stroke in older patients. But serum levels of total *P*CS was independently significantly associated with co-morbidity of PAOD in younger patients. All these results showed that serum total levels of *P*CS were highly associated with atherosclerosis-related disease such as CAD, stroke, and PAOD in CKD patients with hemodialysis. But the association between the serum levels of total IS and co-morbidity of CAD, stroke, and PAOD is lacking in the study. Some studies found that serum levels of IS in hemodialysis patients is associated with markers related to atherosclerosis [4]. Because of the controversial results, the exact association between serum levels of IS and atherosclerosis-related comorbidity in CKD patients need further evaluation.

The study had several limitations. The free-form *P*CS and IS levels were not checked. Furthermore, the mechanism of the higher total-form *P*CS in the DM patients could not be determined. However, this is an important study to evaluate the pro-inflammatory cytokines and protein-bound uremic toxins in hemodialysis patients. In addition, this study found that serum total *P*CS levels were significantly higher in hemodialysis patients with comorbidity of CAD and DM than those without co-morbidity of DM and CAD. This gave us the clue that development of CVD in hemodialysis patients with co-morbidity of DM maybe associated higher total *P*CS due to particular dietary habits and also DM-related alternation in colonic protein fermentation. Besides, due to the constraints of this study design (cross-sectional design), we are not able to provide a powerful evidence for the correlation between serum levels of IS or *P*CS with survival. However, Wang et al. had found association between high total *p*-cresol sulfate and major adverse cardiac events in CKD patients [25]. Wu et al. also showed that serum free *p*-cresol sulfate levels predict cardiovascular and all-cause mortality in elderly hemodialysis patients [17]. All of the results strongly proved the association between cardiovascular disease- related clinical outcome and serum levels of *P*CS in CKD patients.

In conclusion, the study showed that uremic toxins including total *P*CS and IS were not associated with pro-inflammatory markers in ESRD patients with hemodialysis. Besides, serum levels of total *P*CS was independently significantly associated with co-morbidity of CAD and DM.
